# Supplementary material for: Rosin Derivatives as a Platform for the Antiviral Drug Design
Source: Molecules. 2021 Jun 23;26(13):3836. doi: 10.3390/molecules26133836 (PMC8270270; doi:10.3390/molecules26133836)
Supplement: Supplementary file 1 [file molecules-26-03836-s001.zip › molecules-1262091-supplementary.pdf]

SUPPLEMENTARY MATERIALS

Biological activity prediction using PASS-online modeling

Table S1. Biological activity prediction of 12-sulfodehydroabietic acid (**I**)

№	Activity	Activity probability Pa	Inactivity probability Pi	Compound
1	Mucomembranous protector	0.930	0.004	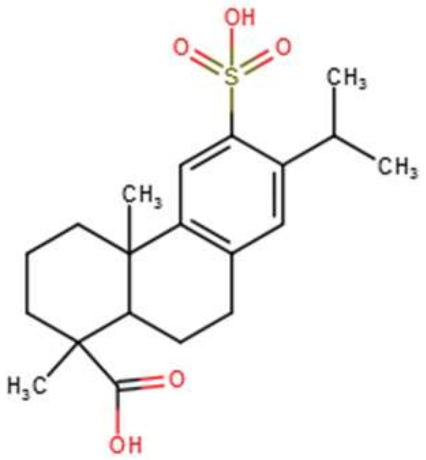
2	Oxidoreductase inhibitor	0.875	0.003	
3	Antihypercholesterolemic	0.853	0.004	
4	Acute neurologic disorders treatment	0.844	0.006	
5	Glyceryl-ether monooxygenase inhibitor	0.826	0.004	
6	Antiviral (Influenza)	0.801	0.003	
7	Membrane permeability inhibitor	0.787	0.012	
8	Alcohol O-acetyltransferase inhibitor	0.757	0.003	
9	Cholestanetriol 26-monooxygenase inhibitor	0.728	0.006	
10	Trans-1,2-dihydrobenzene-1,2-diol dehydrogenase inhibitor	0.726	0.003	
11	Testosterone 17beta-dehydrogenase (NADP+) inhibitor	0.751	0.039	
12	Vasoprotector	0.719	0.009	

TableS2. Biological activity prediction of potassium 12-bromodehydroabietate (**III**)

№	Activity	Activity probability Pa	Inactivity probability Pi	Compound
1	Mucomembranous protector	0.928	0.004	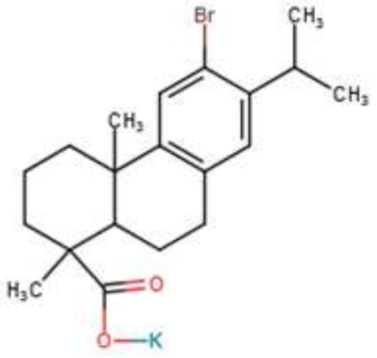
2	Testosterone 17beta-dehydrogenase (NADP+) inhibitor	0.814	0.022	
3	CYP2J substrate	0.791	0.023	
4	Antiviral (Influenza)	0.702	0.005	

Table S3. Biological activity prediction of potassium 12-sulfodehydroabietate (**IV**)

№	**Activity**	**Activity probability Pa**		**Inactivity probability Pi**		**Compound**	
1	Mucomembranous protector	0.976	0.969	0.002	0.002	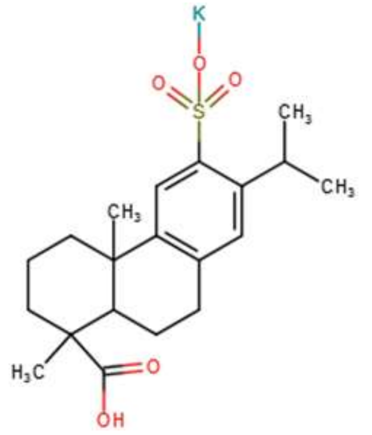	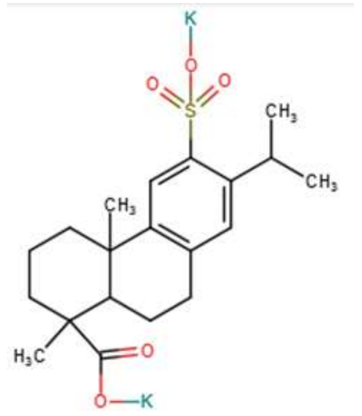
2	Urease inhibitor	0.846	0.755	0.001	0.002		
3	Gastrin inhibitor	0.710	-	0.004	-		
4	Testosterone 17beta-dehydrogenase (NADP+) inhibitor	0.722	-	0.048	-		

TableS4. Biological activity prediction of 12-bromodehydroabietic acid **(X)**

№	Activity	Activity probability Pa	Inactivity probability Pi	Compound
1	Mucomembranous protector	0.959	0.003	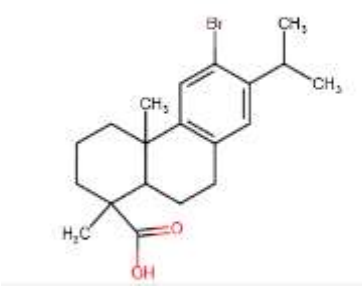
2	Testosterone 17beta-dehydrogenase (NADP+) inhibitor	0.859	0.013	
3	Oxidoreductase inhibitor	0.800	0.006	
4	CYP2J substrate	0.805	0.020	
5	Antiviral (Influenza)	0.768	0.003	
6	Antieczematic	0.725	0.038	

Table S5. Biological activity prediction of abietinol (**XI**) (Pa > 0.7)

№	**Activity**	**Activity probability Pa (%)**	**Inactivity probability**
1	Alkenylglycerophosphocholine hydrolase inhibitor	0.836 (**84**)	0.013
2	Oxidoreductase inhibitor	0.820 (**82**)	0.005
3	Antihypercholesterolemic	0.811 (**81**)	0.005
4	Transcription factor stimulant	0.788 (**79**)	0.003
5	Transcription factor NF kappa B stimulant	0.788 (**79**)	0.003
6	CYP2J substrate	0.794 (**79**)	0.022
7	Mucomembranous protector	0.784 (**78**)	0.023
8	Antieczematic	0.768 (**77**)	0.026
9	Hepatoprotectant	0.724 (**72**)	0.006
10	Alkylacetylglycerophosphatase inhibitor	0.723 (**72**)	0.017
11	Antineoplastic	0.717 (**72**)	0.023
12	Testosterone 17beta-dehydrogenase (NADP+) inhibitor	0.711 (**71**)	0.051
13	Caspase 3 stimulant	0.706 (**71**)	0.011

- The prediction results are presented to the user in the form of a list of names of activity probable types with estimated probabilities of the presence (Pa) and absence of each type of activity (Pi). They have values from 0 to 1. These probabilities are calculated independently from subsamples of active and inactive compounds, and therefore their sum is not equal to one. Pa and Pi are interpreted as estimates of the measure of a substance belonging to the classes of active and inactive compounds, respectively, or as estimates of errors of the first and second kind. The higher the Pa value for a particular activity and the lower the Pi value, the greater the chance of detecting this activity in the experiment.

Revealing the drug-like properties of compounds using SWISS-ADME web server

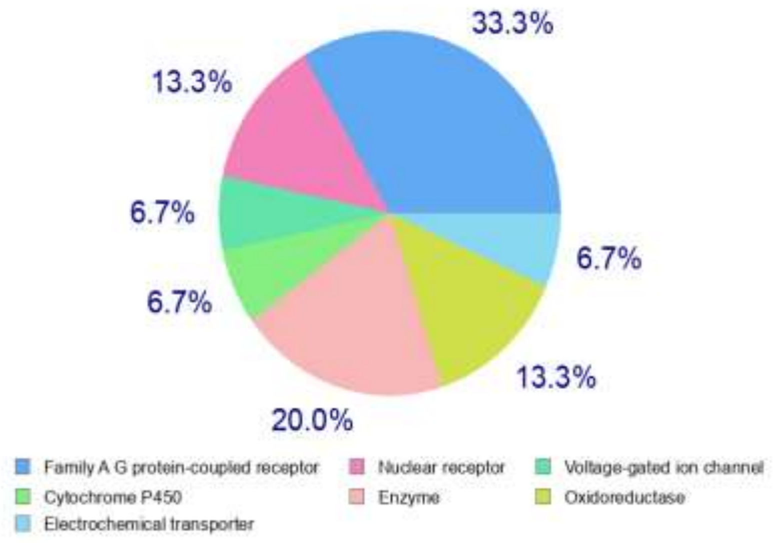

Figure S1. Target proteins - top 15 most likely activities of 12-sulfodehydroabietic acid (**I**)

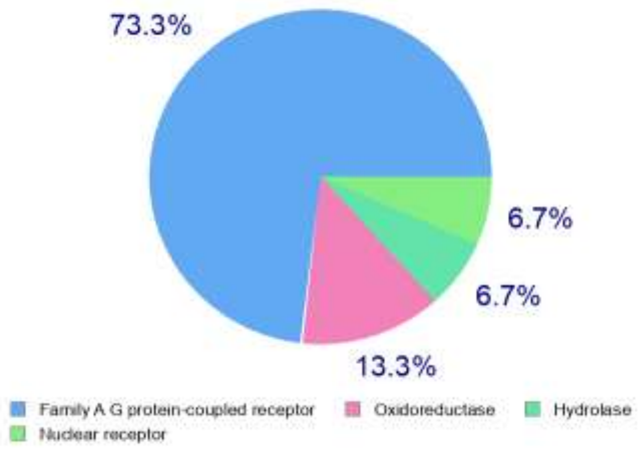

Figure S2. Target proteins - top 15 most likely activities of potassium 12-bromodehydroabietate (**III**)

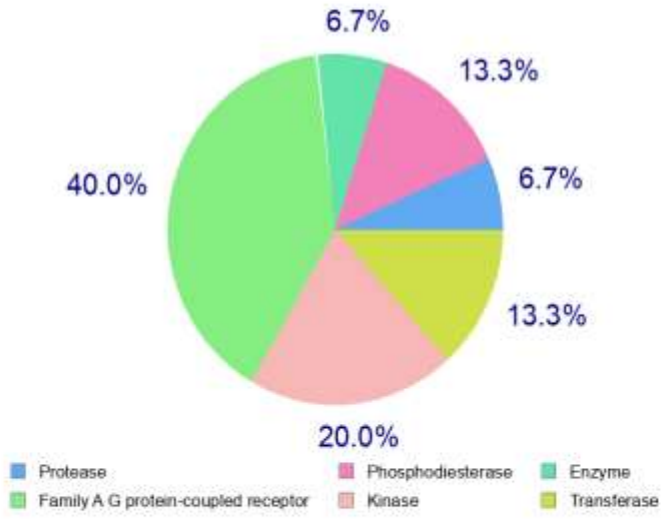

Figure S3. Target proteins - top 15 most likely activities of potassium 12-sulfodehydroabietate (**IV**)

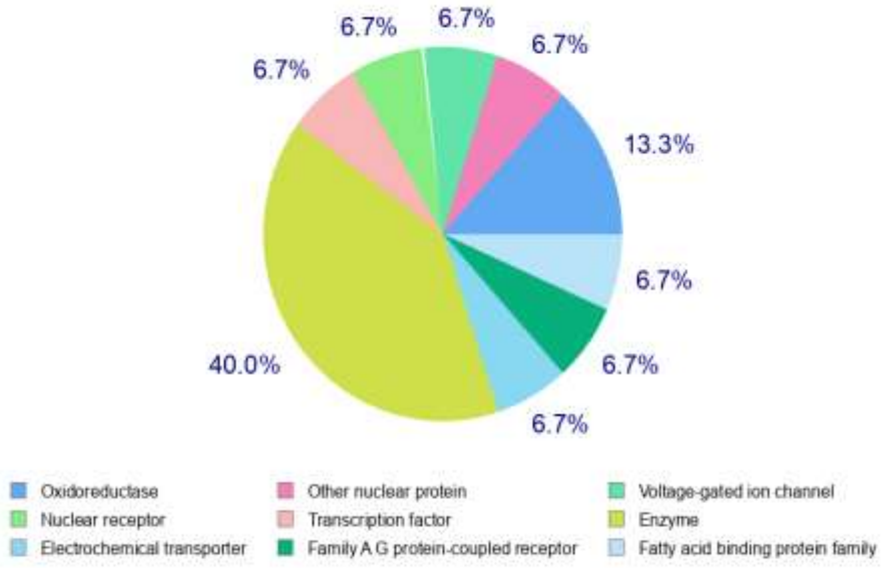

Figure S4. Target proteins - top 15 most likely activities of 12-bromodehydroabietic acid **(X)**

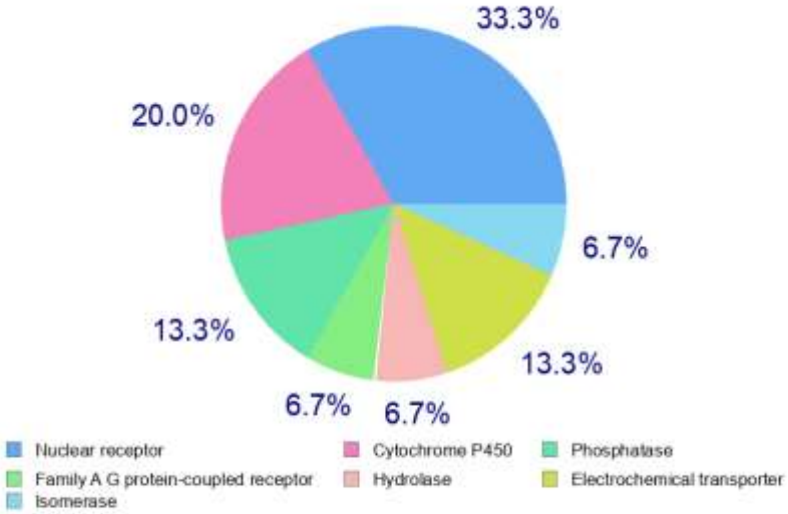

Figure S5. Target proteins - top 15 most likely activities of abietinol (**XI**)
